# Metastasis ability, genomic profile, subtype characteristic and curative efficacy of multiple pulmonary hematogenous metastases in lung cancer

**DOI:** 10.1002/ctm2.1639

**Published:** 2024-03-26

**Authors:** Liuer He, Xin Nie, Jiayi Gao, Yue Yuan, Xue Wang, Xu Li, Fengzhu Guo, Min Tang, Ping Zhang, Lin Li

**Affiliations:** ^1^ Department of Oncology Beijing Hospital, National Center of Gerontology, Institute of Geriatric Medicine, Chinese Academy of Medical Sciences Beijing China; ^2^ Graduate School of Peking Union Medical College Beijing China

Dear Editor,

Metastasis is the primary cause of cancer progression. Hematogenous metastasis is the main pathway, and the rich blood supply of the lungs makes them the most common site. Research on multiple pulmonary hematogenous metastases (MPHM) is lacking.

We investigated hematogenous and lymphatic metastasis ability, gene mutations, subtypes and curative efficacy of MPHM in Non‐small cell lung cancer(NSCLC). We enrolled 123 and 117 patients in MPHM and control groups, respectively (Table S1 and Figure 1). The MPHM group was younger (*p* = 0.018); the proportions of females (*p* = 0.036) and adenocarcinomas (*p* = 0.012) were higher (Table S2). In MPHM, carcinomatous lymphangitis (CL, *p* = 0.004), extrathoracic lymph node metastasis (ELNM, *p* = 0.015), brain metastasis (BM, *p* = 0.019) and multiple extrathoracic organ (≥3) metastases (MEOM, *p* = 0.010) were more common. Tier I gene mutations were more common in MPHM (*p* < 0.001), accompanied by more EGFR (*p* = 0.002) and KRAS (*p* = 0.035) mutations. CL and EGFR and KRAS mutations positively correlated with MPHM (Table S3).

**FIGURE 1:**
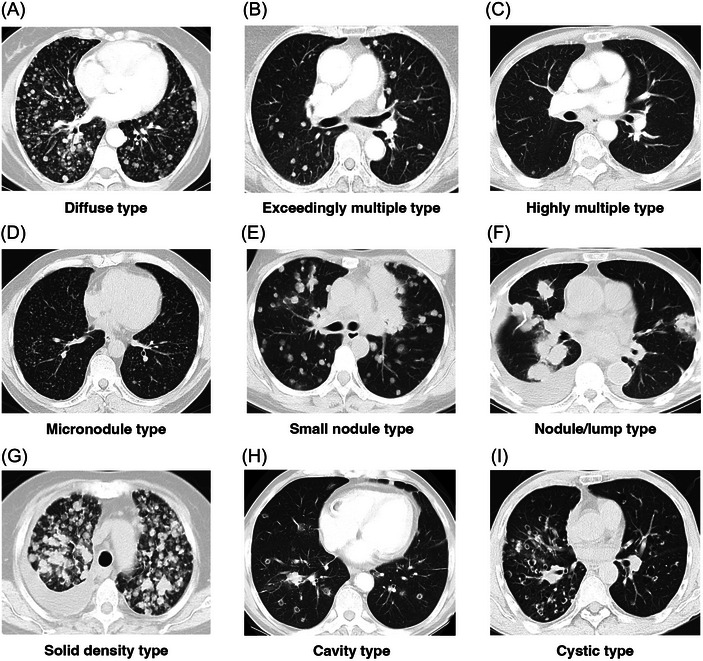
CT imaging of multiple pulmonary hematogenous metastases and subtype of MPHM. (A) Diffuse type: nodules are diffusely distributed in lung. (B) Exceedingly multiple type: more than 10 nodules which are not diffusely distributed. (C) Highly multiple type: more than three but less than or equal to 10 nodules. (D) Micronodule type: 0< maximum diameter ≤5 mm. (E) Small nodule type: 5 mm< maximum diameter ≤10 mm. (F) Nodule/lump type: 10 mm< maximum diameter. (G) Solid density type: the components of the nodules are all solid. (H) Cavity type: the centre of the nodule has formed a cavity. (I) Cystic type: tumour grow along the cyst cavity of original emphysema. MPHM, multiple pulmonary hematogenous metastases.

EGFR and TP53 were the most commonly mutated genes in the MPHM and control groups, respectively (Figure 2A,B). Point mutation was the most common in both (Figure 2C,D). G > A and G > T substitutions were the most common single‐nucleotide variations in the MPHM and control groups, respectively (Figure 2E,F). We analyzed the distribution of variation types for nine common mutations (Figure 2G,H). As vascular endothelial growth factor (VEGF) mediates both hematogenous and lymphatic metastases,^1^ we screened patients who met VEGF measurement criteria (Table S4); the baseline VEGF level in MPHM was higher (*p* = 0.001, Figure 2I,J).

**FIGURE 2:**
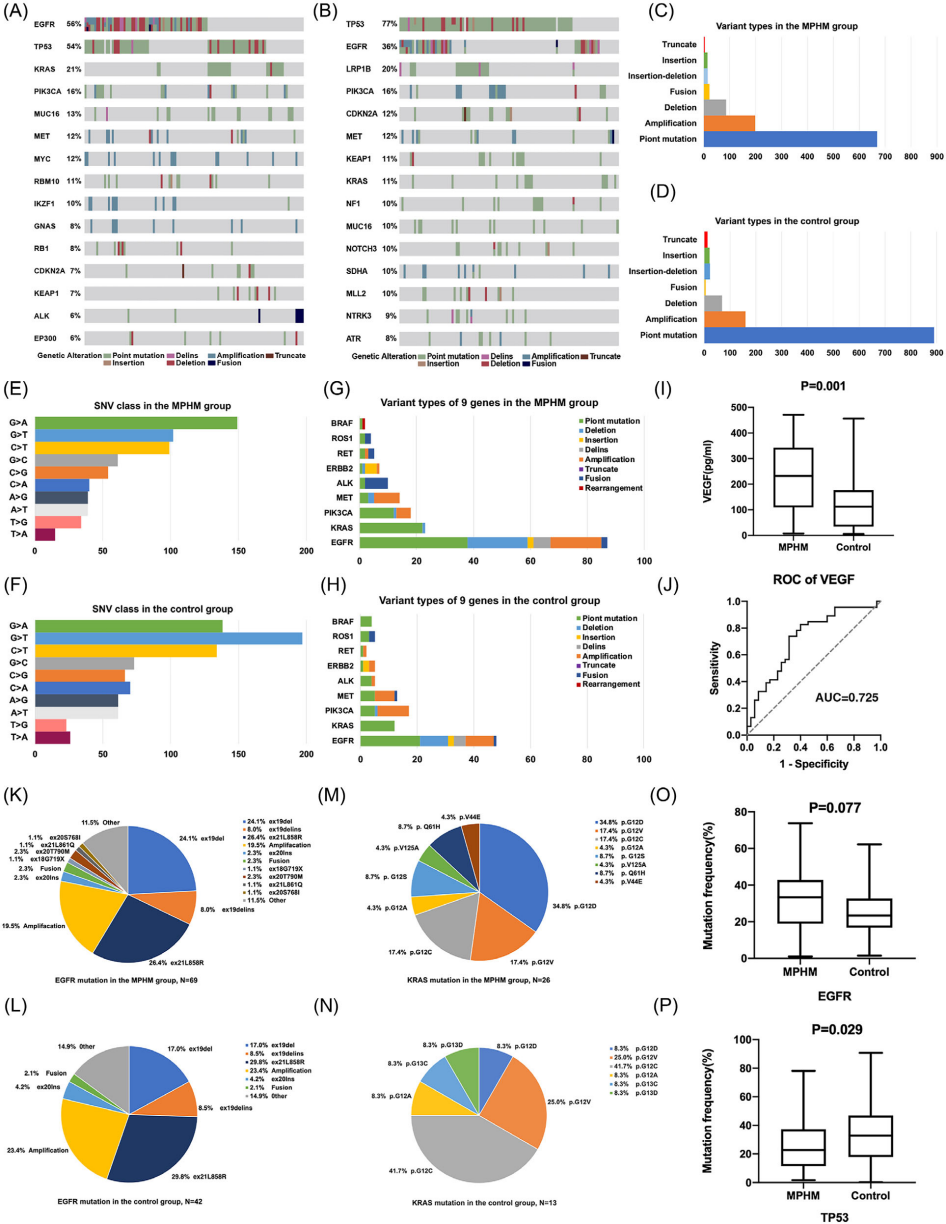
Gene mutation profile in MPHM group and control group. (A,B) Gene mutation profile of top 15 mutated genes in the MPHM group and control group. (C,D) Variation types of gene mutation in the MPHM group and control group. (E,F) Single nucleotide variations of gene in the MPHM group and control group. (G,H) Variation types of nine common mutated genes in the MPHM group and control group. (I) VEGF levels in two groups. (J) ROC curve of VEGF. (K,L) EGFR mutation types in the MPHM group and control group. (M,N) KRAS mutation types in the MPHM group and control group. (O,P) Gene mutation frequencies of EGFR and TP53. MPHM, multiple pulmonary hematogenous metastases; ROC curve, receiver operating characteristic curve; VEGF, vascular endothelial growth factor.

Since EGFR, TP53 and KRAS were the most commonly mutated genes in MPHM, we compared the variant types of EGFR and KRAS mutation (Figure 2K–N). EGFR mutation frequency was higher in MPHM (*p* = 0.077, Figure 2O); that of TP53 was lower (*p* = 0.029, Figure 2P). Other common gene mutations are shown in Figure S1A–D,I–N. Tier I mutation in patients with squamous cell carcinoma and synchronous double tier I mutations were more common in MPHM (Figure S1E–H).

Hematogenous metastasis occurs in three stages: (1) tumour cells infiltrate the vascular system, (2) tumour cells migrate to the pre‐metastatic site and (3) tumours grow after colonization.^2^ We classified MPHM into diffuse, exceedingly multiple and highly multiple types (Figure 1A–C); diffuse‐type tumours could better form metastatic foci as they can colonize almost anywhere in the lung and form abundant metastatic lesions. We classified MPHM into micronodule, small nodule and nodule/lump types (Figure 1D–F); small nodule/nodule/lump (S/N/L) type tumours had stronger post‐colonization growth, as their huge metastatic lesion volume is based on continuous cell proliferation and tumour blood vessel nutritional supply. EGFR mutations were more common in the diffuse type (*p* = 0.056; Figure 3A). MET abnormalities were more common in the S/N/L group (*p* = 0.066; Figure 3B). TMB differed between the diffuse and exceedingly/highly multiple types (*p* = 0.015, Figure 3C). There were no differences in PD‐L1 TPS between the subgroups of 66 patients with baseline TPS (Figure S2A). Comparing CL, ELNM, BM and MEOM among subgroups, ELNM was more common in the micronodule type (Figure 3D), and there was no difference between the diffuse and other types (Figure S2B). Owing to the exceedingly low proportion of cavity and cystic types of MPHM (Figure 1H,I), they were not analyzed. Several patients had diffuse metastatic ground‐glass nodules (Figure S2G), while mixed‐density nodules were more common in synchronous multiple primary lung cancers.^3^

**FIGURE 3:**
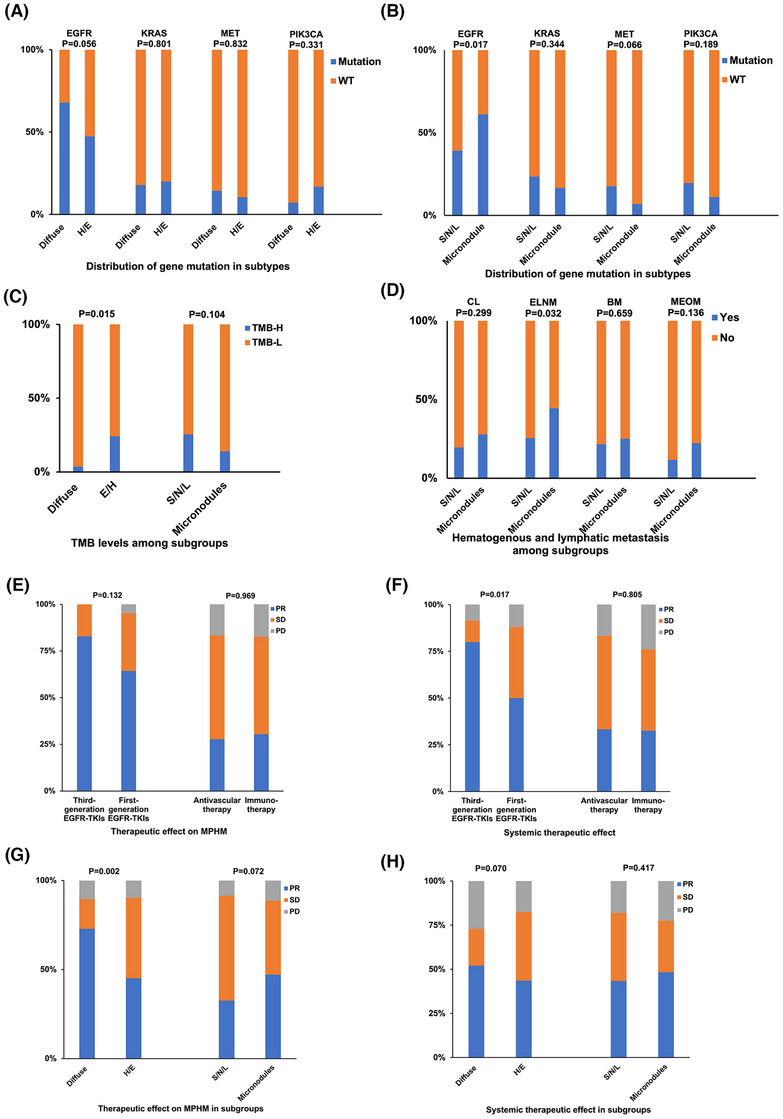
Subtype feature and curative efficacy of MPHM. (A) Distribution of gene mutation among subtypes of MPHM grouped by quantity. (B) Distribution of gene mutation among subtypes of MPHM grouped by volume. (C) Level of TMB among subtypes of MPHM. (D) Comparison of hematogenous and lymphatic metastasis ability between subtype grouped by volume. (E) The curative effect of four therapies on MPHM. (F) The systemic curative effect of four therapies. (G) Curative efficacy on MPHM in subgroups. (H) Systemic curative efficacy on MPHM in subgroups. BM, brain metastasis; CL, carcinomatous lymphangitis; ELNM, extrathoracic lymph node metastasis; H/E, highly/exceedingly multiple type; MEOM, multiple extrathoracic organ metastasis; MPHM, multiple pulmonary hematogenous metastases; S/N/L, small nodule type/nodule type/lump type.

The kappa test revealed a high consistency between the MPHM and systemic efficacies (*p* < 0.001, Table S5). To improve the accuracy of curative effect results, we included patients with baseline genetic testing using qPCR (*N* = 172). We evaluated four main therapeutic regimens in the MPHM group (Tables S6 and S7); third‐generation EGFR‐TKIs showed the best overall and MPHM efficacies (Figure 3E,F). The efficacies of antivascular therapy and immunotherapy were similar. The overall (*p* = 0.002) and MPHM efficacies (*p* = 0.070) of the diffuse type were better (Figure 3G,H). Bone metastasis and MPHM were the main causes of progression after EGFR‐TKIs (Figure S2C,D) and immunotherapy/antivascular therapy, respectively (Figure S2E,F).

Patients in MPHM group tend to be younger, possibly because older patients often have comorbid conditions such as hypertension and hyperlipidemia which alter microvessel wall permeability. The high probability of BM suggests that MPHM cells are more likely to cross the blood–brain barrier. EGFR mediates tumour cell metastasis,^4^ in accordance with its abundant mutation variety and high mutation frequency in MPHM. EGFR mutation was associated with a higher nodule number, and MET abnormality was related to a larger nodule volume. Many studies on hematogenous metastasis in NSCLC have explored post‐colonization growth inhibition. Clinical trials related to BM are common. The CROWN study (*N* = 76) found that loratinib improved the intracranial objective response rate (iORR) (64.9% vs. 17.9%, *p* < 0.001) compared to clotozantinib. Patients with an intracranial response during 3 years of treatment accounted for 29% in loratinib group and 0% in clotozantinib group.^5^ The OCEAN study revealed that among 39 patients receiving osimertinib, the iORR reached 66.7% and median intracranial progression‐free survival (iPFS) reached 25.2 months.^6^ The GAP BRAIN study (*N* = 161) found that gefitinib plus chemotherapy improved the iORR (85.0% vs. 63.0%, *p* = 0.002) and median iPFS (15.6  vs 9.1 months, *p* < 0.001) compared to gefitinib alone.^6^ The CAP‐BRAIN study revealed that among 45 patients receiving camrelizumab, pemetrexed and carboplatin, the iORR reached 52.5%, and median iPFS reached 7.6 months.^7^ Several studies have focused on suppressing formation of metastatic lesions. The Impower150 study found that in 802 stage IV patients, atelizumab, bevacizumab, carboplatin and paclitaxel (ABCP) therapy resulted in a lower probability of developing new brain metastases in subsequent treatments compared to ACP therapy (7% vs. 12%, *p* = 0.017).^8^ ECOG‐ACRIN E1505 revealed that in 1501 stage IB–IIIA patients, chemotherapy plus bevacizumab lead to a lower probability of developing new brain metastases in subsequent treatments compared to chemotherapy (6% vs. 10%, *p* = 0.02).^9^

This study is the first to explore MPHM. In our study, MPHM had stronger ability for hematogenous and lymphatic metastasis, accompanied by higher probability, variety and frequency of EGFR mutation. EGFR mutation was associated with a higher nodule number, and MET abnormality was related to a larger nodule volume. Third‐generation EGFR TKI showed the best therapeutic effect on MPHM.

## AUTHOR CONTRIBUTIONS

All authors participated in the planning and execution of this study or analysis of the study data. Liuer He and Lin Li designed this study. All authors collected the data and conducted the relevant experiments. Liuer He and Xin Nie performed the statistical analyses. Ping Zhang and Min Tang performed all the pathological evaluation. Liuer He, Xu Li and Xin Nie reviewed the CT. Liuer He drafted the manuscript. Jiayi Gao, Yue Yuan, Xue Wang and Liuer He made the figures. Liuer He, Fengzhu Guo and Lin Li revised the manuscript. All authors read and approved the final version of the manuscript. Lin Li are corresponding authors of this manuscript.

## CONFLICT OF INTEREST STATEMENT

The authors declare no conflicts of interest.

## FUNDING INFORMATION

National High Level Hospital Clinical Research Funding, Grant/Award: BJ‐2023‐073; CAMS Innovation Fund for Medical Sciences, Grant/Award: 2021‐I2M‐1‐012

## ETHICS STATEMENT

This study was approved by the Ethical Committee of Beijing Hospital.

## Supporting information

Supporting Information

Supporting Information
Gene mutation types in MPHM group and control group. (A–B) PIK3CA mutation types in the MPHM group and control group. (C–D) MET mutation types in the MPHM group and control group. (E–F) Tier I gene mutations in the lung squamous cell carcinoma patients from the MPHM group and control group. (G–H) Dual tier I mutation in the MPHM group and control group. (I–N) Gene mutation frequencies or copy number of KRAS, MET, PIK3CA, CDKN2A, KEAP1, MUC16 in two groups. MPHM, multiple pulmonary hematogenous metastases.

Supporting Information
(A) Level of PD‐L1 TPS among subtypes of MPHM. (B) Comparison of hematogenous and lymphatic metastasis ability between subtype grouped by quantity. (C) Cause of progression after applification of third generation EGFR TKI. (D) Cause of progression after applification of first generation EGFR TKI. (E) Cause of progression after applification of anti‐vascular therapy. (F) Cause of progression after applification of immunotherapy. (G) Mixed density subtype of MPHM.

## Data Availability

All the data and materials are available upon reasonable request from the corresponding author.
