# Supplementary material for: Metastasis ability, genomic profile, subtype characteristic and curative efficacy of multiple pulmonary hematogenous metastases in lung cancer
Source: Clin Transl Med. 2024 Mar 26;14(3):e1639. doi: 10.1002/ctm2.1639 (PMC10964915; doi:10.1002/ctm2.1639)
Supplement: Supplementary file 1 — Supporting Information [file CTM2-14-e1639-s001.docx]

| \| Table S1  Inclusion and exclusion criteria for patients with MPHM \| \| \| --- \| --- \| \| Inclusion Criteria \| Exclusion Criteria \| \| 1.Pathological diagnosis of NSCLC \| 1.With other tumor species \| \| 2.Patients who consulted Beijing Hospital from December 2021 to September 2023 \| 2.With synchronous multiple primary lung cancers \| \| 3. With baseline next-generation sequencing (1021-gene panel/654-gene panel/571-gene panel) \| 3. Receive systematic treatment or had stopped systematic treatment for less than 6 months before being diagnosed with stage IV NSCLC \| \| 4. Stage IV NSCLC patients with more than 3 pulmonary hematogenous metastases diagnosed through PET/CT or CT assisted by Yizhun Intelligence AI system  (MPHM group) \| 4. Stage IV NSCLC without MPHM through PET/CT or CT assisted by Yizhun Intelligence AI system  (control group) \|   Table S2 Baseline characteristics of 240 NSCLC patients | | | |
| --- | --- | --- | --- | --- | --- | --- | --- | --- | --- | --- | --- | --- | --- | --- | --- |
|  | MPHM Group (n=123) | Control Group (n=117) | P |
| Age (median [IQR]) | 67 [32, 88] | 69 [37, 92] | 0.018 |
| Gender (%) |  |  | 0.036 |
| Female | 54 (43.9) | 36 (30.8) |  |
| Male | 69 (56.1) | 81(69.2) |  |
| Pathological type (%) |  |  | 0.012 |
| Adenocarcinoma | 91 (74.0) | 68 (58.1) |  |
| Squamous carcinoma | 20 (16.3) | 38 (32.5) |  |
| Other | 12 (9.8) | 11 (9.4) |  |
| Carcinomatous lymphangitis |  |  | 0.004 |
| Yes | 30 (24.4) | 12 (10.3) |  |
| No | 93 (75.6) | 105（89.7） |  |
| Extrathoracic lymph nodes metastasis |  |  | 0.015 |
| Yes | 45 (36.6) | 26 (22.2) |  |
| No | 78 (63.4) | 91 (77.7) |  |
| Liver metastasis |  |  | 0.997 |
| Yes | 21 (17.1) | 20 (17.1) |  |
| No | 102 (82.9) | 97 (82.9) |  |
| Bone metastasis |  |  | 0.953 |
| Yes | 50 (40.6) | 48 (41.0) |  |
| No | 73 (59.3) | 69 (59.0) |  |
| Brain metastasis |  |  | 0.019 |
| Yes | 29 (23.6) | 14 (12.0) |  |
| No | 94 (76.4) | 103 (88.0) |  |
| Adrenal metastasis |  |  | 0.842 |
| Yes | 19 (15.4) | 17 (14.5) |  |
| No | 104 (84.6) | 100 (85.5) |  |
| Multiple extrathoracic organ (≥3) metastases |  |  | 0.010 |
| Yes | 22 (17.9) | 8 (6.8) |  |
| No | 101 (82.1) | 109 (93.2) |  |
| EGFR |  |  | 0.002 |
| Yes | 69 (56.1) | 42 (35.9) |  |
| No | 54 (43.9) | 75 (64.1) |  |
| KRAS |  |  | 0.035 |
| Yes | 26 (21.1) | 13 (11.1) |  |
| No | 97 (78.9) | 104 (88.9) |  |
| MET |  |  | 0.957 |
| Yes | 15 (12.2) | 14 (12.0) |  |
| No | 108 (87.8) | 103 (88.0) |  |
| PIK3CA |  |  | 0.997 |
| Yes | 20 (16.3) | 19 (16.2) |  |
| No | 103 (83.7) | 98 (83.8) |  |
| Tier I gene mutation |  |  | <0.001 |
| Yes | 92 (74.8) | 52 (44.4) |  |
| No | 31 (25.2) | 65 (55.6) |  |
| TP53 |  |  | <0.001 |
| Yes | 66 (53.7) | 90 (76.9) |  |
| No | 57 (46.3) | 27 (23.1) |  |
| TMB-H |  |  | 0.003 |
| Yes | 22 (17.9) | 40 (34.2) |  |
| No | 101 (82.1) | 75 (65.8) |  |
| Tumor markers (median [IQR]) |  |  |  |
| CEA | 14.55 [1.10, 964.50] | 8.80 [0.60, 914.10] | 0.503 |
| SCC | 0.8 [0.2, 70] | 1.0 [0.3,123.8] | 0.249 |

| Table S3 Regression analysis of 240 NSCLC patients | | | | | |
| --- | --- | --- | --- | --- | --- |
| Variable | Multiple Pulmonary Hematogenous Metastases | | | | |
|  | Univariate Analysis | |  | Multivariate Analysis | |
|  | HR [95%CI] | P |  | HR [95%CI] | P |
| Age, y | 0.973 [0.950, 0.996] | 0.020 |  | 0.988 [0.962, 1.014] | 0.327 |
| Gender (male vs female) | 0.568 [0.334, 0.965] | 0.036 |  | 0.731 [0.392, 1.364] | 0.319 |
| Pathological subtype (adenocarcinoma vs other) | 2.049 [1.188, 3.535] | 0.010 |  | 1.007 [0.514, 1.974] | 0.984 |
| Carcinomatous lymphangitis (yes vs no) | 2.823 [1.367, 5.830] | 0.005 |  | 3.428 [1.527, 7.695] | 0.003 |
| Extrathoracic lymph nodes metastasis (yes vs no) | 2.019 [1.142, 3.570] | 0.016 |  | 1.847 [0.975, 3.408] | 0.060 |
| Liver metastasis (yes vs no) | 0.999 [0.510, 1.956] | 0.997 |  |  |  |
| Bone metastasis (yes vs no) | 0.985 [0.588, 1.648] | 0.953 |  |  |  |
| Brain metastasis (yes vs no) | 2.270 [1.131, 4.554] | 0.026 |  | 1.824 [0.844, 3.940] | 0.126 |
| Adrenal metastasis (yes vs no) | 1.075 [0.529, 2.185] | 0.842 |  |  |  |
| Multiple extrathoracic organ metastases (yes vs no) | 2.968 [1.264, 6.966] | 0.012 |  |  |  |
| EGFR mutation (yes vs no) | 2.169 [1.287, 3.658] | 0.002 |  | 2.332 [1.181, 4.605] | 0.015 |
| KRAS mutation (yes vs no) | 2.121 [1.007, 4.470] | 0.038 |  | 3.773 [1.549, 9.189] | 0.003 |
| TP53 mutation (yes vs no) | 0.478 [0.281, 0.813] | <0.001 |  | 0.573 [0.313, 1.047] | 0.070 |
| TMB-H (yes vs no) | 0.408 [0.224, 0.741] | 0.004 |  | 0.535 [0.260, 1.098] | 0.088 |

| Table S4 Inclusion and exclusion criteria for baseline measurement of VEGF | |
| --- | --- |
| The included patients (N=36) | The excluded patients (N=45) |
| 1.Patients from two groups who measured VEGF before systematic treatment | 1. Angiodysplastic diseases including asthma, diabetes retinopathy, liver disease, etc; |
|  | 2. Current infections unrelated to anti-tumor therapy; |
|  | 3. Other inflammatory diseases include ulcerative colitis, rheumatoid arthritisc etc; |
|  | 4. Received surgery within 2 months; |
|  | 5. Recovery period of myocardial infarction. |

| Table S5 Consistency of efficacy | | | | | |
| --- | --- | --- | --- | --- | --- |
|  |  | Systemic efficacy | | |  |
|  |  | PR | SD | PD | Total |
| MPHM efficacy | PR | 70 | 7 | 14 | 91 |
|  | SD | 9 | 51 | 4 | 64 |
|  | PD | 0 | 0 | 17 | 17 |
|  | Total | 79 | 58 | 35 | 172 |

| Table S6 Efficacy of MPHM | | | | | | |
| --- | --- | --- | --- | --- | --- | --- |
|  | N | PR | SD | PD | ORR | DCR |
| Third-generation EGFR-TKIs | 35 | 29 | 6 | 0 | 82.9% | 100% |
| First-generation EGFR-TKIs | 42 | 27 | 13 | 2 | 64.3% | 95.2% |
| Chemotherapy+Antivascular therapy | 18 | 5 | 10 | 3 | 27.8% | 83.3% |
| Chemotherapy+Immunotherapy | 46 | 14 | 24 | 8 | 30.4% | 82.6% |

| Table S7 Systemic efficacy | | | | | | |
| --- | --- | --- | --- | --- | --- | --- |
|  | N | PR | SD | PD | ORR | DCR |
| Third-generation EGFR-TKIs | 35 | 28 | 4 | 3 | 80.0% | 91.4% |
| First-generation EGFR-TKIs | 42 | 21 | 16 | 5 | 50.0% | 88.1% |
| Chemotherapy+Antivascular therapy | 18 | 6 | 9 | 3 | 33.3% | 83.3% |
| Chemotherapy+Immunotherapy | 46 | 15 | 20 | 11 | 32.6% | 76.1% |
