# Supplementary figures and images for: Metastasis ability, genomic profile, subtype characteristic and curative efficacy of multiple pulmonary hematogenous metastases in lung cancer
Source: Clin Transl Med. 2024 Mar 26;14(3):e1639. doi: 10.1002/ctm2.1639 (PMC10964915; doi:10.1002/ctm2.1639)

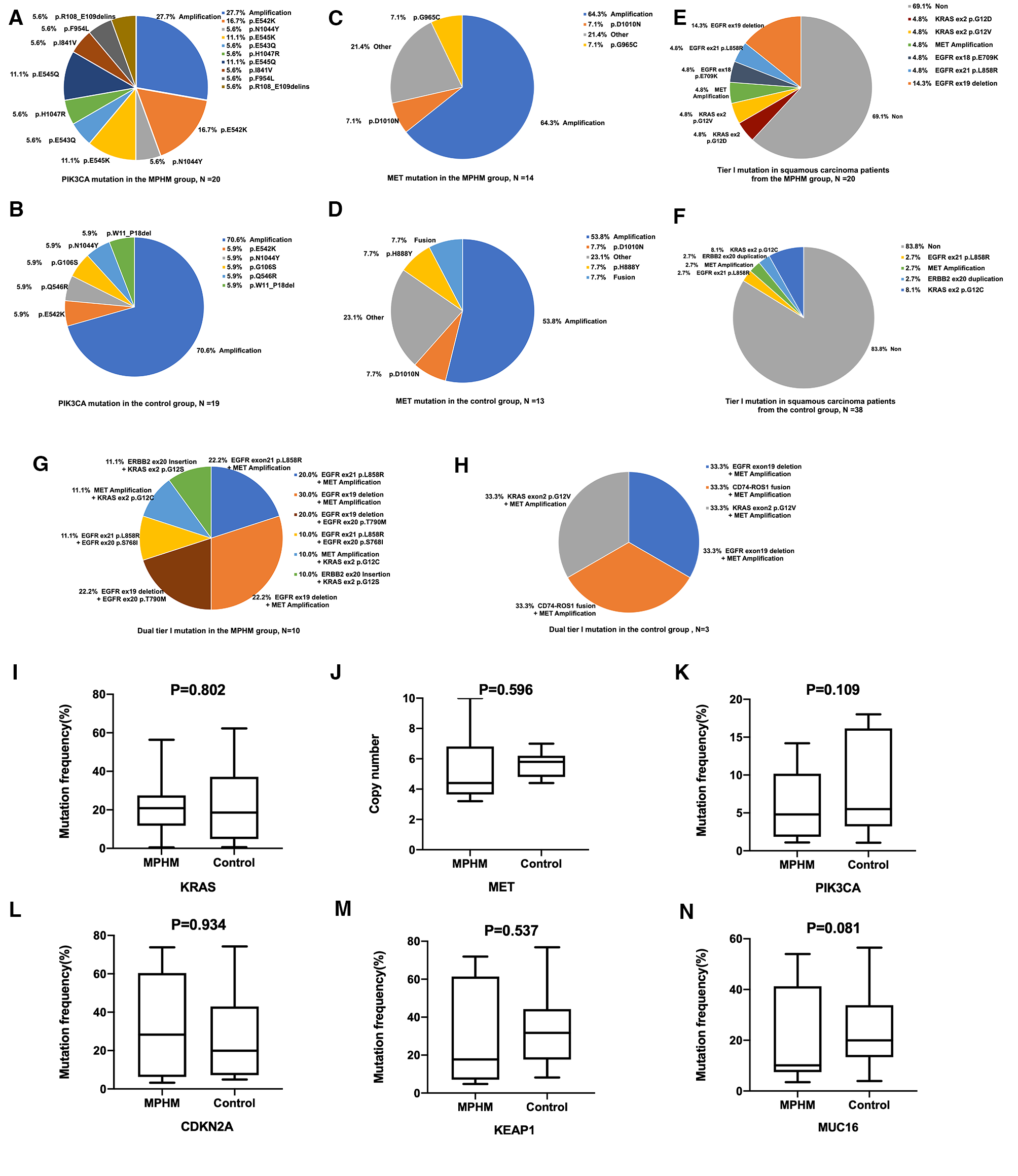

Supplement: Supplementary file 2 — Supporting Information Gene mutation types in MPHM group and control group. (A–B) PIK3CA mutation types in the MPHM group and control group. (C–D) MET mutation types in the MPHM group and control group. (E–F) Tier I gene mutations in the lung squamous cell carcinoma patients from the MPHM group and control group. (G–H) Dual tier I mutation in the MPHM group and control group. (I–N) Gene mutation frequencies or copy number of KRAS, MET, PIK3CA, CDKN2A, KEAP1, MUC16 in two groups. MPHM, multiple pulmonary hematogenous metastases. [file CTM2-14-e1639-s003.tif]

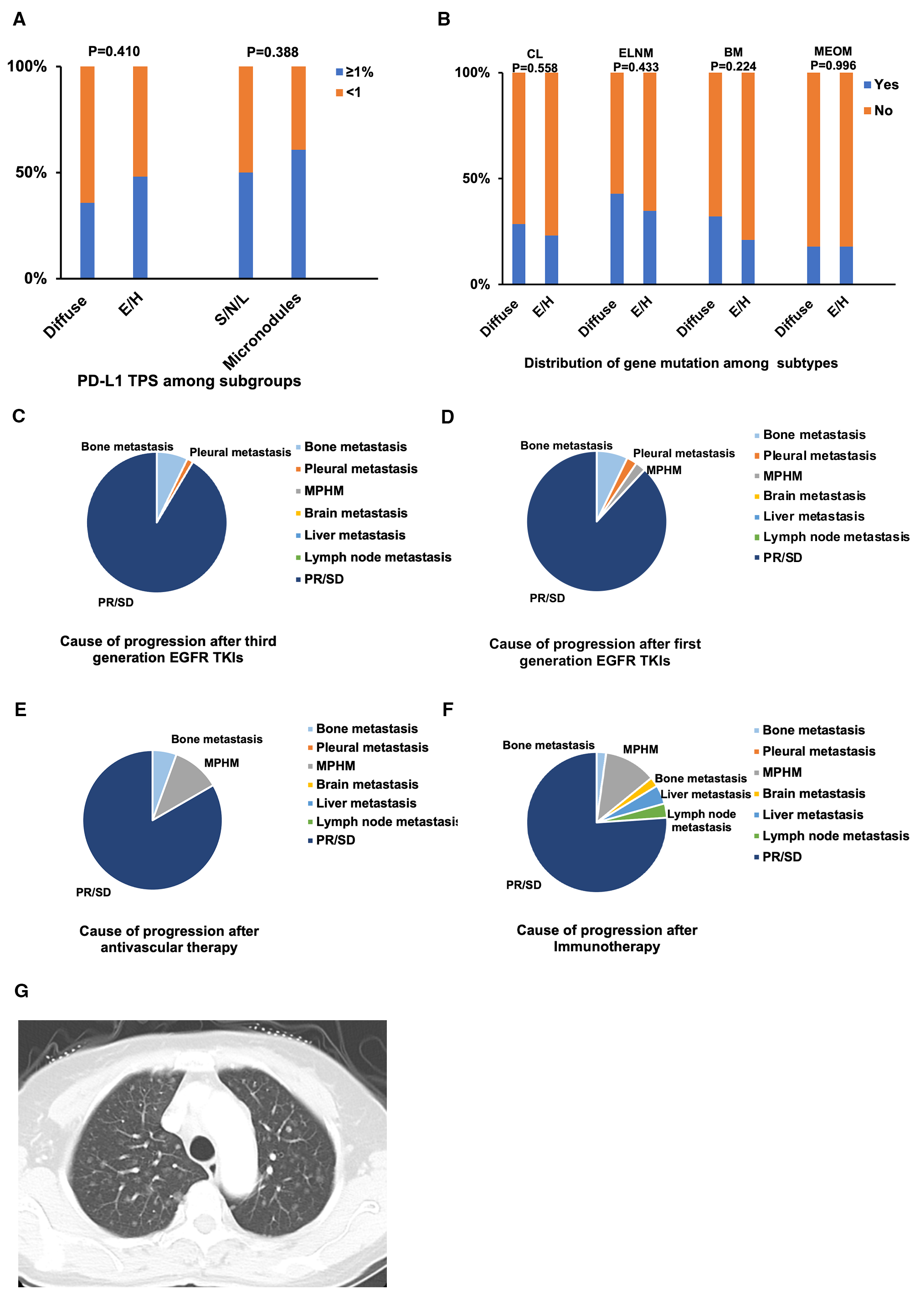

Supplement: Supplementary file 3 — Supporting Information (A) Level of PD‐L1 TPS among subtypes of MPHM. (B) Comparison of hematogenous and lymphatic metastasis ability between subtype grouped by quantity. (C) Cause of progression after applification of third generation EGFR TKI. (D) Cause of progression after applification of first generation EGFR TKI. (E) Cause of progression after applification of anti‐vascular therapy. (F) Cause of progression after applification of immunotherapy. (G) Mixed density subtype of MPHM. [file CTM2-14-e1639-s002.tif]
